# Digital Technologies, Ethical Questions, and the Need of an Informational Framework

**DOI:** 10.1007/s13347-018-0326-2

**Published:** 2018-08-10

**Authors:** Federica Russo

**Affiliations:** Department of Philosophy, University of Amsterdam, Oude Turfmarkt 141-147, 1012GC Amsterdam, Netherlands

**Keywords:** Digital technologies, Philosophy of information, Technological determinism, Poiesis

## Abstract

Technologies have always been bearers of profound changes in science, society, and any other aspect of life. The latest technological revolution—the digital revolution—is no exception in this respect. This paper presents the revolution brought about by digital technologies through the lenses of a specific approach: the philosophy of information. It is argued that the adoption of an informational approach helps avoiding utopian or dystopian approaches to (digital) technology, both expressions of technological determinism. Such an approach provides a conceptual framework able to address the ethical challenges that digital technologies pose, without getting stuck in the dichotomous thinking of technological determinism, and to bring together ethics, ontology, and epistemology into a coherent account.

## The Digital Revolution

The telephone has changed communication. From the moment it was invented in the course of the nineteenth century, we could exchange news and information more quickly and keep in touch more frequently, even at long distance. Today, with the smartphone, we can do many things, *including* phone calls. Two objects often called with the same name—*phone*—well represent the transition from the analogue to the digital. What did it change, exactly, from the old telephone to the smartphone?

Technology, or rather, its evolutions, has been often used to draw the line in history between “before” and “after.” For instance, Arendt (1958) proposed to classify civilizations through the use of specific technological objects (e.g., we speak of the stone age and now of the digital age); Wyatt (2007) argued that the categorization is not only temporal, but also cultural (e.g., windmills characterize the Netherlands and watches Switzerland); Mumford (1961) suggested that this tendency to associate entire millennia with a specific artifact comes from archeology and anthropology, as we try to understand human beings and society through the artifacts used in specific epochs. Information and Communication Technologies (ICTs) do mark a radical change, in these three senses. Arguably, though, the most relevant change brought about by ICTs concerns the way in which *information* is transmitted and processed. Floridi (2016) reconstructs these changes by identifying three macro-periods: (i) pre-history, (ii) history, and (iii) hyper-history. The information cycle (occurrence, transmission, process and management, use of information) has seen important changes through time: in the pre-history of the ICTs, we could not record information, as knowledge was passed on orally. With the advent of writing, we entered history, or the information age. Today, with the advent of digital technologies, we have entered *hyper*-history. The difference between history and hyper-history, however, is not only in the quantity and speed of information transmitted, but especially in *how* it is transmitted and processed. Thus, Lévy (1997) rightly speaks of a “speed of evolution of knowledge” and of a “collective intelligence,” formed by the social existences of the subjects who participate in it, and which has the capacity to continually reshape itself. These ideas need to be complemented with that of *connective* intelligence (De Kerckhove 1998), which is not based on what we have learned, but on how we can connect things to each other.

With these connections, digital technologies do something that no technology has been able to do before: they transform the surrounding environment and create new ontological spaces. In these environments, technologies can interact with one another, and sometimes without our intermediation. Social media, big data, or e-health technologies are all examples of ICTs that are thought to raise new ethical problems or dilemmas. In this commentary, I explain why the ethical issues raised by ICTs, in order to be properly addressed, are in need of a different conceptual framework, notably an informational approach. Simply put, I argue that before raising new ethical challenges, ICTs bring about profound changes at the ontological and epistemological level. In Section 2, I frame the discussion on digital technologies as a problem related to technological determinism; here, contributions in the literature oscillate between utopian and dystopian visions, both entailing specific normative positions. Therefore, in Section 3, I reconstruct traditional approaches to technology and ethics and argue that they do not offer us the right tools to understand and address the profound changes brought about by digital technologies and to escape technological determinism. Finally, in Section 4, I delineate the contours of an informational approach, able to address the ontological, epistemological, and ethical challenges posed by digital technologies.

## Technological Determinism

Digital technologies often create opposite reactions. Some have seen in them unprecedented improvements (e.g., Negroponte 1995), while others have predicted catastrophic scenarios (e.g., Rifkin 2004). Both utopian and the dystopian visions take for granted that the introduction of ICTs in our lives and in society puts us on a pre-defined path, which we cannot move away from. Digital technologies thus become *normative*, in a positive or negative sense. This normative dimension of technology, and a fortiori of ICTs, makes the link between digital technologies and ethics explicit: technology is closely related to the sphere of action and of decision. A number of authors, for instance Radder (2009) and, before him, Winner (1980), explained why technological artifacts are intrinsically “political” and “ethical.” For example, the decision to build a bridge is not politically neutral because it can foster communication, social relations, and trade between two places or communities. But the way in which the bridge is built could also disadvantage other parts. According to Winner, the bridge in Long Island (New York) is an icon of the political character of the artifacts. Technologies, Radder further argues, are closely linked to the environment in which they operate. This environment is not only material but also social and cultural. Technologies are therefore also linked to the norms that regulate how people interact, or who is allowed to interact with whom, and what the technologies should or should not do. The arguments of Radder and Winner are set in general terms with respect to technology and remain valid, a fortiori, for ICTs. Just think of how digital technologies influence social relations, of how they are used in politics (at the time of writing, the scandal of Cambridge Analytica is a case in point), and of how digital technologies themselves are the object of political decisions.

A *first strategy* to get out of technological determinism is to think in terms of *possibilities*. The idea comes already from Wiener (1948, 1950), who posed the ethical question with extremely lucid tones. Cybernetics, says Wiener, offers *possibilities*, for better or for worse, and we must therefore pay attention to which possibilities we decide to develop or not to develop. He further argued that technology is not so much an applied science as an *applied social and moral philosophy*. In his work, Wiener (1950) expressed concerns about the possible military use of cybernetics. His idea was that cybernetics should be at the service of human beings, not developed for their exploitation or harming them. A similar line of argument is to be found in Lyotard (1979). He argued that while new technologies led to caesuras, and specifically to the crisis of the “metanarrative,” digital technologies also bear *opportunities*. The computerization of society can be a way to exercise control and regulate the market system. Yet, we are not simply condemned to suffer the effects of digitalization but, proactively, we can exploit them to improve our condition. And analogously, digitalization can be a way to stimulate and facilitate the collective discussion of certain norms. The possibilities, or *affordances*, related to what artifacts (including ICTs) allow us to *do*—are also explored in ecological psychology and then further elaborated in design studies and media studies (Gibson 2015; Norman 2013; Bucher and Helmond 2018). In Section 4, we shall see that instead of being passive victims of (digital) technology, we *create* technology and the material, conceptual, or ethical environments, possibilities, or affordances for its production or use; this makes us also *responsible* for the space of possibilities that we create.

A *second strategy* to avoid technological determinism is to rethink the relations between science and technology or between knowledge and its applications. It is widely believed that technology, and in our case digital technology, raises ethical questions for its *applied* character. With science, we study high-energy physics, but it is with the technology that we build the atomic bomb. Therefore, according to this line of reasoning, science is not good or bad and right or wrong; these judgments are instead directed to technology, which is believed to be an *application* of science. However, it is important to note that the separation between technology and science is not as clear-cut. Douglas (2014) cogently argues that this distinction, as well as the one between “pure” and “applied” science, is a product of our historical, philosophical, sociological reflection on techno-scientific practices. Douglas further argues that what over the years has been deemed as “pure” or “applied” depended not only on the methods, objects, or purpose of the techno-scientific activity at stake, but also on whether scientists, in given historical moments and embedded in specific socio-political contexts, reclaimed autonomy in their research, for instance with respect to pre-determined goals fixed by governments or other political authorities. More to the point, science and technology are, especially nowadays, sets of practices in which our intelligence and actions intertwine with complex technological systems and apparati, in equally complex socio-political contexts (Russo 2016). We hold therefore a responsibility for, and a *poietic* power on, the technology that we produce and create—I shall come back to this point in Section 4.

Dorrestijn (2017) also reconstructs the tensions between utopian and dystopian views in philosophy of technology. He argues that while “technological mediation” is an important step to move beyond technological determinism (see e.g. Latour (1992) or Verbeek (2011)), Foucault’s “ethics as art of living” offers us better instruments to address the question of ethics in technology (Foucault 1988, 1990a, b, 1997). After investigating how “disciplinary power” limits our freedom, Foucault’s later work on ethics was devoted to understanding how we *cope* with these external influences and to develop an ethics in which human beings *strive to have mastery* on their own lives. Thus Dorrestijn’s reading of Foucault’s ethics also seems to point to the same direction as the previously identified strategies: what needs investigation is the interaction between technology and humans in *creating* space of possibilities and the ethical assessment that stems from the *actions* involved in these environments—Section 4 aims precisely to offer a conceptual framework for this.

Both these strategies to get out of technological determinism point to the same direction: the question of normativity arises not so much, or not only, because (digital) technologies are intrinsically good or bad or because they are an application of some “pure” science. The ethical question arises because technology (and science) has to do with *action*. We act when we publish a picture on Facebook, when we analyze samples in the laboratory, when we build and implant an artificial limb following an amputation, and also when we give consent to nuclear research, or stem cells. In these, and many other actions, human beings, groups of human beings, institutions, and artifacts (digital and otherwise) are involved in given decision-making processes.

## Traditional Ethical Approaches to Technology

### Technology, Science, and Ethics

Mitcham and Briggle (2009) reconstruct the relations between technology and ethics, from antiquity to the present day. They argue that from the Greeks we have learned to recognize the specificity of technologies, and at the same time, we have inherited the idea that what pertains to the téchne is often morally inferior to knowledge (and, a fortiori, to the science that produces it). This is possibly at the basis of the idea that ethical questions about technology originate in their applied character. From the Greeks, we also inherited the idea that, somehow, natural objects are more “real” than artifacts. This idea was strengthened by the introduction of digital technologies and by the alleged dichotomy between “online” and “offline,” which is problematized (and defused) in Section 4. Thus, the Greeks did give ethical and political value to the téchne, but not to the epistème. This is important for the second “escape route” to get away from technological determinism: rethinking the relations between science and technology (or between knowledge and its applications). The dominant narrative in history of (philosophy of) science emphasizes the innovations in scientific methods brought about by Galilean and Baconian methods, and that led to the unquestionable success of contemporary science. But these narratives also gloss over the fact that Galilean and Baconian science is highly technologized and politicized. It is a science in which the invention and the production of technological artifacts lead to know, to better understand, and to control nature—in sum, it leads to improving on the human condition. The “techno-science” of Galileo or Bacon, therefore, has already a political, ethical, and social value. Just think of the *New Atlantis*, in which Bacon imagines a happy society in which science and technology are at the service of the common good. Science and technology thus have, for Galileo and Bacon, a high ethical and political value. A position reinforced in the Enlightenment and, subsequently, during the industrial revolution: science and technology are at the service of human beings to satisfy their desires and wills. This is also the period in which a “science of ethics” emerges, viz., a systematic discourse that gives the foundations, or the principles, to decide what is right, useful, or good.

During the first half of the twentieth century, neo-positivist scientists and philosophers reintroduced the idea of a separation between technology (subject to moral assessment) and science (not subject to moral assessment)—their discourse on science and scientific method was largely a-moral (nevertheless, many of them were politically and socially engaged). A purely theoretical analysis of scientific method leads to the idea that science is not good or bad, but its applications are. This explains why the applications of science, or technology, have been mainly discussed in relation to the socio-political context or the personal-existential sphere, while epistemological and metaphysical discussions about science have been largely developed independently of ethical discourse. I argue, instead, that the ethical discourse on digital technologies *must* recover a unity, an explicit connection with the fundamental issues related to knowledge and metaphysics. To do this, we must go beyond classical approaches and find a conceptual framework that allows us to hold together science and technology on the one hand and ethics, epistemology, and metaphysics on the other. This is the challenge that in recent years has been taken up by the philosophy of information and that I present in Section 4.

### Socio-political-existential Critiques and Analytic Ethics

In the broad area of philosophy of technology and critical theory, three main approaches can be identified, namely, the socio-political critique, the critical-existential critique, and analytic ethics. Notwithstanding their fundamental contributions to investigating the effects of technologies, they do not offer us the right tools to address the new challenges posed by ICTs, or so I argue.

The socio-political and socio-existential critiques start from the premise that technology profoundly changes the human condition. In particular, these schools aim to explain two phenomena: de-humanization and inauthenticity. The socio-political critique, specifically, aims to reform the economic and political structures associated with technology, in order to re-establish an essential condition of human beings: freedom. Within this area of research, the criticism of Marx and Engels (1872) is best known. The optimism that came from Bacon and from the Enlightenment, and according to which the development of science and technology will be for the benefit of humanity, is followed by a dystopian vision. Technology changes society and the economic structures that precede the industrial revolution become obsolete. This leads to devastating effects at the social level: power is badly distributed and technology (i.e., the industrial revolution) leads to oppression and alienation. A notable position against the Marxist argument that technology creates oppression comes from Arendt (1958) who, in developing her view on the *vita activa*, emphasizes the characteristic of homo *faber* in our human condition. In Section 4, we shall see how Arendt’s view resonates with the concept of poiesis, as developed within an informational approach, which can get us out of a Marxist type of technological determinism.

The Frankfurt School shifts the axis of discussion from economics to culture. Marcuse (1964), for example, questions the relationship between technology and social relations, trying to understand whether the former influences the latter, or vice versa. In the first case, in fact, we would be confronted with a clear argument in favour of technological determinism. But it is far from obvious that this influence be unidirectional, rather than bidirectional. Feenberg (2002), for one, claims that technology is neither decisive nor neutral. Somehow, Feenberg provides an answer to Marcuse’s question: the influence is *not* unidirectional, but decidedly *bi*directional. This is a delicate issue that can get us out of the impasse between embracing technological determinism, often accompanied by strongly dystopian visions, or embracing a progressive and linear vision, according to which progress (here, technological progress) invariably brings improvements—a clear Whiggish stance. Both these visions do not correspond to reality. In fact, technological innovations may induce change in society and in our habits and needs, but sometimes the opposite is the case (see e.g. Vincenti 1993; Pinch and Bijker 1984). In Section 4, we shall see how an informational approach can account for the mutual influence between technology and its users and/or producers *and* for the profound ontological transformations that ICTs brought about.

The socio-existential critique takes up the presuppositions of the socio-political critique but emphasizes the relationship between technology and the meaning of life—a topic further elaborated in contemporary post-phenomenology (see e.g. Rosenberger and Verbeek (2015)). Science and technology are not only artifacts but also “forms of consciousness” that therefore have a real impact on the existential dimension. Famously, Heidegger (1954) saw technology as a form of truth. In his view, science and technology are intertwined practices. Heidegger, in a way, overcomes the Greek dichotomy between epistème and téchne by giving technology the power to “unveil” the truth, a possibility that was previously given only to the epistème, to science. As we shall see in Section 4, this is important because technology contributes to *creating* new environments. Yet, we need more: next to the poietic character of technology, we must be able to address normative questions that stem from it. It will be argued that an informational approach is able to do that.

Finally, analytic ethics focuses on specific problems that originate in the use of technology and aims to develop targeted topics, clarifying the concepts involved. Winkler and Coombs (1993) distinguish three distinct subfields within ethics: bioethics, business ethics, and environmental ethics, to which we nowadays add a fourth area, namely, computer ethics. Computer ethics provides standards of behavior for IT developers and users (think of the decalogue proposed as early as 1992 by the Computer Ethics Institute, or of the code of ethics of the Association for Computing Machinery). Today’s challenges specifically concern digital technologies (e.g., the possibility of a democratic technology, the Internet as a vehicle for the dissemination of information but also of child pornographic material, privacy issues, or the “digital divide”). The analytic method is supported by a case-by-case approach or “casuistry” (Toulmin and Jonsen 1988). While recurring themes may be identified (e.g., justice and equity lead to questions about health and safety, information, privacy, and risk; autonomy and freedom lead to worries about well-being and health or welfare and, consequently, to concerns about the environment and sustainability), ethical analyses are here limited to specific cases, without any attempts to generalize or to unify the problems in a single conceptual framework, be it ethical, epistemological, or ontological. As we shall see in Section 4, an informational approach provides tools to reconnect these different dimensions.

In sum, all the aforementioned traditional approaches have the merit of having grasped the main problematic aspects of technology: its role in society and politics, in the personal sphere, and its very specific aspects, for example in biotechnology or in the field of privacy. Very often (and especially in the socio-political and socio-existential approach) discussions are driven by a fear of technology, and dystopian visions seem to take over. With the advent of digital technologies, many of the problems identified several decades ago have been further exacerbated. This literature has greatly contributed to the in-depth understanding of technology, but has provided comparatively fewer tools for dealing with *new* questions posed by new technologies. Somehow, we found ourselves unprepared to face a radical change posed by the digital revolution, because in dissecting the impact of technology on the political, social, or personal sphere, an overall framework has not been developed or got lost: we lack appropriate conceptual tools to deal with ethical problems that originate in changes that are ontological and epistemological in the first place. An attempt in this direction is given by the philosophy of information, which I introduce next.

## The Informational Approach: Digital Technologies and Ethics

### The Re-Ontologization of the Real

As already mentioned in Section 1, Floridi (2013, 2016) argues that, with the advent of digital technologies, we entered *hyper*-history. Not only because we are now able to exchange and process large amounts of information at unprecedented speeds, but because we are witnessing a transformation that is simultaneously epistemological and ontological. Digital technologies are not just transforming the political or existential spheres or creating specific ethical problems. These transformations are the effect of a more radical change: digital technologies are capable of changing the environment or, using a neologism of Floridi, they *re-ontologize* the real.

It is worth clarifying right away that the philosophy of information does not support or presuppose technological determinism. It is not a question of utopia or dystopia, of imagining technological paradises or hells, but to understand *what* changed. Much emphasis is given to new ethical challenges, such as privacy or cyber-security, but these challenges emerge precisely because digital technologies have brought about changes that are ontological and epistemological in the first instance, with implications at the ethical level. Therefore, before discussing the ethics of information, we must take a step back. What does the digital revolution entail?

*Information* is the basic concept for understanding the surrounding world, ourselves, and the relationship between the world and us. Information can be (i) a *resource*, when we use it, for example, to make decisions, or it can be (ii) a *product*, when we use it to generate other information and then modify the surrounding environment, i.e., information is conceived as (iii) a *target*. By *infosphere*, we mean the totality of the perceivable, phenomenal, and informational environment (simply put, reality as a whole) and with inforgs those organisms capable of processing information (animals, humans, machines). In the infosphere, we find the old libraries but also digital libraries, and there are lawns, trees, animals, computers, human beings, institutions, etc.

The concept of inforg is key as it does not mark an “essential” distinction between organism (whether humans or animals) and machines. The ability of processing information belongs to both humans and computers and, today, to smartphones too. This theme had been anticipated in the *Cyborg Manifesto* of Haraway (1985). The fundamental question posed by Haraway is what would distinguish human beings from other species or from machines. This question is not new, but now more than ever timely (see, e.g., the work of Turkle (2014) on digital technologies and the formation of the self). Haraway’s critique is to show that any holistic or essentialist approach cannot account for what human beings are. The cyborg manifesto is a clear rejection of technological determinism, because social relationships have an impact on technology. A feminist critique thus helps us shed light on how sources of power, socio-economic structures, and socio-technical systems are intertwined. Such a reflection, that starts with problematizing the role of women in the production of knowledge, imagination, and the practice of technologies, shows that there is no essence to be found, but there are, however, *relationships* to be understood—a position, this, also in line with post-phenomenology. There isn't, in other words, any essential distinction between “natural” and “artificial” or between human beings and machines—a bedrock of the philosophy of information.

In general, re-ontologizing means to design a system or to fundamentally transform the *nature* of a system. This is what ICTs do, and that Floridi explains with the concept of “in-betweeness,” namely, what stands in between humans and technologies. There are three forms of interactions in which technology interposes between a user and a “prompter,” that is, what stimulates, or suggests, the use of a certain technology: (i) *human-technology-nature*, (ii) *human-technology-technology*, and (iii) *technology-technology-technology*. In the second and in the third type, ICTs have the power of altering the environment. In the third one, specifically, human beings are outside the chain of dependence and interaction. Digital technologies do something different than just boosting (e.g., a hammer) or increasing (e.g., a washing machine) human abilities or capabilities. They change the way in which we relate to the surrounding reality, or the infosphere, to others inforgs and ourselves.

From the ontological point of view, there are two important ideas. *First*, in the transition from analogue to digital, there is a progressive convergence of digital *resources* and digital *tools*. In duplicating a document with the old mimeograph, there was a technology that produced a *new* copy of the document. Today, the Word document I am typing is a digital technology, produced by another digital technology (that is, the word-processor software). *Second*, digital technologies change the degree of *ontological friction* (i.e., what contrasts the flow of information in a given region of the infosphere). Digital technologies often decrease ontological friction, thus creating information overflows or information surplus. However, more information does not necessarily mean *better* information. This is why the discourse on ICTs cannot be dichotomous: utopian or dystopian. Changes in ontological friction can do anything along the whole spectrum of *possibilities* or affordances: from greatly improving to badly worsen a given situation. They can help or hinder information flow in various ways, and this can have important ethical repercussions, as we shall see later. In turn, an ethical assessment cannot be issued via the binary reasoning typical of technological determinism, but always requires a much more nuanced analysis.

### New Ontological Environments, Poiesis, and Constructionist Ethics

Arguably, the biggest transformation provoked by the digital revolution is the creation of a hybrid dimension between online and offline. Digital technologies have re-ontologized reality by creating a dimension that the philosophers of information have called *onlife* (Floridi 2015): neither online nor offline, but both at the same time. We must therefore come to terms with a *hybrid* ontological dimension. Lévy had anticipated the concept of onlife, saying that life is not only reality but also possibility, actuality, and virtuality. Lévy has, overall, an optimistic position regarding the potential of ICTs. Before him, it is perhaps Simondon (1958) the philosopher who, well before the advent of digital technologies, understood how technological objects “become concrete” and, interacting with the environment and with human beings, are themselves subject to development and to change. Again, we are faced with intermediate positions with respect to utopian or dystopian scenarios, and that call for a deep understanding of the relationships between human beings and machines and between natural and artificial objects, avoiding technological determinism.

In the philosophy of information, we find a further argument to undermine technological determinism, namely *poiesis*. We are not “victims” of technology, but we *create* technology, in the first place. As anticipated earlier, (technological) production is the results of *actions*, and for this reason, what happens in the infosphere must be subjected to the examination of ethical analysis.

The Greek concept of poiesis originally applied to the creation of artifacts, but it should be extended to shed light to an essential dimension of human beings. The human dimension has been discussed in relation to our being *Homo sapiens*, *ludens*, or *faber*. But our ability to *create* is in need of special attention. For one, Arendt (1958) emphasizes the activities of making and fabricating as essential parts of the *vita activa*. There is a connection between knowledge, which is part of the contemplative life, and doing: we can know what we can do, and here, the technological tools, or instruments, for the production of knowledge and experiments play a fundamental role. Arendt thus establishes a link between knowing and doing: it is not just that we aim to know “what” or “why” something is, but also *how* it is so-and-so. But we can achieve knowledge of “how” only insofar as we believe in our “productive capacities,” in using instruments, setting up experiments, and therefore gaining knowledge through making. However, our productive, or poietic, capacities are not limited to artifacts or instruments. Just as we create objects, we create concepts or situations—*homo poieticus* is also the techno-scientist and the philosopher, insofar has both contribute to the production of material or conceptual tools to understand and act upon the world around us (Russo 2012).

The notion of poiesis helps us bridge ontology/epistemology and ethics, in the following way. With “moral agent,” we refer to any agents in the infosphere subject to an ethical assessment; among moral agents, we include, in addition to us human beings, also artificial agents. The central idea behind information ethics is that any informational agents—be they human, artificial, or hybrid—are *proactive* agents that create objects, concepts, or situations. It is therefore necessary to develop a construc*tionist* (not constructivist) ethics in which all moral agents (humans, groups of humans, human-like, or super-humans, etc.) can be responsible for the situations they engender (Floridi 2013). To repeat, we are not victims of technologies, but we instead *create* the environments, possibilities, or affordances, subject to ethical evaluation.

Often, the traditional approaches mentioned in Section 3, and especially analytic ethics, have emphasized ethical dilemmas. This is the framework of an “action-based” ethics, in which the reasoning pertains to a *given*, specific situation. However, we ought to ask a more fundamental question, often not taken into account: how did we find ourselves in such a situation? The central point of information ethics is that inforgs do not simply react to a situation, but they proactively create it. In an informational approach, we look at the *source* of moral action. Another important feature of constructionist ethics is that it is not centered on the *single* moral agent, but can instead deal with moral problems at the group level. In fact, an ethical discourse must be able to deal with different levels: the individual inforg (ego-poiesis), groups of inforg (socio-poiesis), or the infosphere (eco-poiesis). We can even think of humanity itself as a moral agent: humanity has a responsibility towards the infosphere, meant as a natural, social, or cultural environment, both present and future.

Information ethics proposes a general model for analyzing ethical problems that may arise at different levels (the moral evaluation of the single inforg, of a group of hybrid or heterogeneous inforgs, and of humanity itself). The analysis does not start from the question what is right or wrong according to one or the other ethical theory, but from a more fundamental fact: ethical problems in the digital sphere are related to the question whether information be a resource, a target, or a product (as explained above). A correct ethical analysis must, first of all, identify the correct *level of abstraction*, that is, (i) whether the problem concerns the information as resource, target, or product, (ii) which moral agents are involved, and (iii) how the inforgs created such a situation. Only at that point, we can ask specific questions about what is right or wrong and good or bad, which admittedly are the ultimate goal of an ethical assessment. Constructionist ethics is not another ethical theory, but a *conceptual framework* for analyzing an ethical problem. The ethical and moral analysis is based on a precise analysis of reality, namely, the aforementioned “onlife” dimension: digital technologies are not a special, apart, problem but they are part and parcel of our reality. If we do not understand this fundamental re-ontologization, we cannot pose any ethical questions in an adequate manner.

Integrating ethical analyses into a global (i.e., ontological and epistemological) understanding of the digital phenomenon allows us to highlight the fundamental difference between human beings and digital artifacts. It is not a question of intelligence or of information processing. What marks the difference between human beings and digital artifacts is our ability and capability to entertain a relation with the world through *specific ethical choices*. We do not have a privileged place in the infosphere because of our ability to process information, but we continue to have a central place because we have a *responsibility* towards all the inforgs and the infosphere, and that we cannot delegate to others. In this sense, constructionist ethic allows us to recover other important perspectives, such as the ethics of hospitality of the aforementioned Lévy or the ethics of the responsibility of Jonas (1979).

## The Need of a Different Conceptual Framework

Digital technologies change reality in a fundamental way. After centuries of scientific studies and of philosophical reflections, we could reasonably claim to master the offline world. But the online world is not just a dimension “added” to the development of digital technologies to be studied in the same way as the offline world. The online dimension has actually led to a confusion and fusion of online and offline. We are *onlife*, to borrow the neologism of the philosophers of information. Problems such as the privacy of personal data on the Internet, or the right to be forgotten, are clearly generated by digital technologies. But their “onlife” character requires that—before any ethical verdict can be advanced—the nature of the problem is understood. Understanding the nature of the problem implies ontological and epistemological reflections that, together, can inform ethics.

An important lesson from the philosophy of information is that there is no essential difference between humans and machines. This has repercussions on a number of debates. On the one hand, we must rethink our categories to understand the nature of informational organisms. What makes us humans distinct from animals or computers is not our ability to process information but the *responsibility* we hold towards other inforgs and the infosphere during the whole information cycle. On the other hand, and at a more general epistemological level, the effects of ICTs are profound and pervasive. The creation of knowledge is not an exclusive prerogative of human beings: knowledge becomes situated, embodied, distributed, and relational—and this across humans, machines, institutions, or environments. Recognizing these characteristics of knowledge help mending (philosophy of) technology and (philosophy of) science so that ethical questions can be an integral part of them.

It is only once we recognize how much the *onlife* world differs from the old offline world that we can sketch out a new discourse about ethics. In particular, an informational approach provides an overarching methodological framework in which we can juggle with three fundamental elements of philosophical thinking: what the world is (i.e., ontology), how we can get knowledge of the world (i.e., epistemology), and the normative dimension at ethico-political level. These three are essentially related and interconnected. However, due to the hyper-specialization of the sciences and of philosophy, they grew into distinct sub-disciplines that, by and large, talk past each other rather than *to* each other. Digital technologies, with their ethical challenges, provide us with the opportunity to bring together ethics, ontology, and epistemology in a coherent approach. To date, the philosophy of information is the approach that more than others has the potential to understand this new *onlife* dimension and to guide us out of the impasse of technological determinism.

## References

[CR1] Arendt, H. (1958). *The human condition*. University of Chicago Press.

[CR2] Bucher, T., Helmond, A. (2018). *The affordances of social media platforms. In The SAGE handbook of social media, by Jean Burgess, Alice Marwick, and Thomas Poell, 233–53*. SAGE Publications Ltd.

[CR3] De Kerckhove, D. (1998). *Connected intelligence: the arrival of the web society*. Kogan Page.

[CR4] Dorrestijn S (2017). The care of our hybrid selves: ethics in times of technical mediation. Foundations of Science.

[CR5] Douglas H (2014). Pure science and the problem of progress. Studies in History and Philosophy of Science.

[CR6] Feenberg, A. (2002). *Transforming technology: a critical theory revisited*. Oxford University Press.

[CR7] Floridi, L. (2013). *The ethics of information*. Oxford University Press.

[CR8] Floridi, L. (Ed.) (2015). *The onlife manifesto.* Being human in a hyperconnected era. Springer.

[CR9] Floridi, L. (2016). *The 4th revolution: how the infosphere is reshaping human reality*. Oxford University Press.

[CR10] Foucault, M. (1988). *Technologies of the Self. In Technologies of the Self. A Seminar with Michel Foucault,* edited by Luther H. Martin, Huck Gutman, and Patrick H. Hutton, 16–49. Tavistock Publications.

[CR11] ———. 1990a. *The Care of the Self: Volume 3 of The History of Sexuality*. Vintage Books ed. New York: Vintage Books.

[CR12] ———. 1990b. *The Use of Pleasure: Volume 2 of The History of Sexuality*. New York: Vintage Books. http://search.ebscohost.com/login.aspx?direct=true&scope=site&db=nlebk&db=nlabk&AN=733100.

[CR13] ———. 1997. The Ethics of the Concern for Self as Practice of Freedom. In *The Essential Works of Foucault, 1954-1984,* edited by Paul Rabinow, translated by James D. Faubion. New York: New Press.

[CR14] Gibson JJ (2015). The ecological approach to visual perception.

[CR15] Haraway D (1985). A manifesto for cyborgs: science, technology, and socialist feminism in the 1980s. Soc Rev.

[CR16] Heidegger, M. (1954). *The question concerning technology and other essays*. 1970th ed. Harper & Row.

[CR17] Jonas H (1979). The imperative of responsibility: In Search of an ethics for the technological age.

[CR18] Latour, B. (1992). Where are the missing masses? The sociology of a few mundane artifacts. In W. E. Bijker and J. Law (Eds.), *Shaping technology/building society: studies in sociotechnical change* (pp. 225–58). MIT Press.

[CR19] Lévy P (1997). L’intelligence Collective: Pour Une Anthropologie Du Cyberspace. La Découverte/Poche 27.

[CR20] Lyotard, J-F. (1979). *La Condition Postmoderne*. Les Editions de Minuit.

[CR21] Marcuse, H. (1964). *One-dimensional man*. Beacon.

[CR22] Marx, K., & Engels, F. (1872). Das Kapital: Kritik Der Politischen Ökonomie. 2016th ed. Hamburg Nikol Verlag.

[CR23] Mitcham, C., & Briggle, A. (2009). The interaction of ethics and technology in historical perspective. In A. Meijers (Ed.), *Philosophy of technology and engineering sciences* (pp. 1147–94).

[CR24] Mumford L (1961). History: neglected clue to technological change. Technology and Culture.

[CR25] Negroponte N (1995). Being digital.

[CR26] Norman DA (2013). The design of everyday things.

[CR27] Pinch TJ, Bijker WE (1984). The social construction of facts and artefacts: or how the sociology of science and the sociology of technology might benefit each other. Social Studies of Science.

[CR28] Radder, H. 2009. Science, technology and the science–technology relationship. In A. Meijers (Ed.), *Philosophy of technology and engineering sciences*, vol.9, (pp. 65–92). Elsevier.

[CR29] Rifkin J (2004). The end of work: the decline of the global labor force and the dawn of the post-market era.

[CR30] Rosenberger R, Verbeek P-P (2015). Postphenomenological investigations: essays on human-technology relations. Postphenomenology and the philosophy of technology.

[CR31] Russo, F. (2012). The Homo poieticus and the bridge between physis and techne. In H. Demir (Ed.), *Luciano Floridi’s philosophy of technology*. *Critical reflections*. Springer.

[CR32] Russo F (2016). On the poietic character of technology. Humana Mente Journal of Philosophical Studies.

[CR33] Simondon, G. (1958). *Du Mode D’existence Des Objets Techniques*. Aubier.

[CR34] Toulmin, S., & Jonsen, A. R. (1988). *The abuse of casuistry: a history of moral reasoning*. University of California Press.

[CR35] Turkle S (2014). Life on the screen.

[CR36] Verbeek P-P (2011). Moralizing technology: understanding and designing the morality of things.

[CR37] Vincenti, W. (1993). *What engineers know and how they know it: analytical studies from aeronautical history*. Johns Hopkins University Press.

[CR38] Wiener, N. (1948). *Cybernetics. Or control and communication in the animal and the machine*. *(1985) ed*. The MIT Press.

[CR39] Wiener, N. (1950). *The human use of human beings. (1989) ed*. Free Association Books.

[CR40] Winkler, E., & Coombs J. R. (Eds.) (1993). *Applied ethics: a reader*. Wiley-Blackwell.

[CR41] Winner L (1980). Do artifacts have politics?. Daedalus.

[CR42] Wyatt, S. (2007). Technological determinism is dead. In E. J. Hackett, O. Amsterdamska, M. L. Lynch, & J. Wajcman (Eds), *The handbook of science and technology studies*, 3rd ed., (pp. 165–80). The MIT Press.

